# 18F-FDG PET/CT guided salvage radiotherapy strategies for lymph-nodal relapses in gynecological cancers: SBRT vs ENRT

**DOI:** 10.1177/03008916251336055

**Published:** 2025-04-18

**Authors:** Andrei Fodor, Martina Midulla, Chiara Brombin, Paola M. V. Rancoita, Alice Bergamini, Paola Mangili, Miriam Torrisi, Lucia Perna, Emanuela Rabaiotti, Italo Dell’Oca, Chiara L. Deantoni, Luca Bocciolone, Claudio Fiorino, Antonella Del Vecchio, Mariaclelia S. Di Serio, Giorgia Mangili, Nadia G. Di Muzio

**Affiliations:** 1 Department of Radiation Oncology, IRCCS San Raffaele Scientific Institute, Milan, Italy; 2 University Center for Statistics in the Biomedical Sciences, Vita-Salute San Raffaele University, Milan, Italy; 3 Vita-Salute San Raffaele University, Milan, Italy; 4 Department of Oncologic Gynecology, IRCCS San Raffaele Scientific Institute, Milan, Italy; 5 Medical Physics, IRCCS San Raffaele Scientific Institute, Milan, Italy

**Keywords:** Lymph node relapse, gynecologic tumors, extended nodal radiotherapy (ENRT), positron emission tomography (PET) guidance, simultaneous integrated boost, stereotactic body radiotherapy

## Abstract

**Objective:**

To identify outcome differences between extended nodal radiotherapy (ENRT) with simultaneous integrated boost (SIB) and stereotactic body radiotherapy (SBRT), performed with advanced radiotherapy techniques, both of which were 18F-Fluoro-Deoxy-Glucose (FDG) PET/CT guided, for lymph-node (LN) relapses of gynecological tumors, and to identify the most important determining factors.

**Methods:**

Records of gynecologic patients treated in a single-institution with FDG PET/CT guided intensity-modulated radiotherapy (IMRT), image-guided radiotherapy (IGRT), or SBRT, were reviewed, and only patients at first salvage radiotherapy for LN relapses were considered. Local relapse-free- (LRFS), regional relapse-free- (RRFS), distant metastasis-free- (DMFS), disease-free-(DFS) and overall-survival (OS), as well as acute and late toxicity (with CTCAE v5.0 score), were determined.

**Results:**

Fifty-eight patients (23 ENRT+SIB; 35 SBRT) treated for 178 LNs from February 2007-April 2023, were identified. Median biological equivalent dose (BED10) delivered to PET-positive LNs was 76.5 Gy (Interquartile range-IQR- 74.4;78.7) for ENRT, and 72 Gy (IQR 59.5;75.6) for SBRT. Median follow-up was 81.1(IQR 48.5; 117.2) and 37.0 (IQR 21.3; 58.4) months for ENRT and SBRT, respectively. Thirty-six-month estimated LRFS was 90.2% for ENRT and 82.6% for SBRT; RRFS was 69% and 63.4%, DMFS 26.1% and 44.3%, and OS 73.7% and 60.4%; no statistically significant differences were found between the two groups (logrank test, p= 0.29). ENRT recorded more acute (p⩽0.033), but not late, toxicities.

**Conclusions:**

ENRT+SIB and SBRT for gynecological LN tumor relapses obtain similar results in terms of disease-free and OS, with fair toxicity. Prospective studies with higher patient numbers are needed.

## Introduction

The best therapeutic strategy for lymph-node (LN) metastases from gynecological tumors remains undefined; the few studies in the literature are heterogeneous for both sample characteristics and treatment techniques, despite recent attempts to better focus analyses according to primary tumor.^1-7^ Oligometastatic disease (OMD) treatment is arousing increased interest after the addition of radiotherapy to standard systemic therapy demonstrated increased survival, and the possibility of delaying some systemic treatments.^8,9^ Prospective studies are lacking for gynecologic tumors, as reported in meta-analyses investigating the role of local treatments in OMD.^10,11^ Fluoro-deoxy-glucose (FDG) positron emission tomography (PET)/computed tomography (CT) has good sensitivity, specificity, and diagnostic accuracy for gynecological cancer LN recurrences,^12-15^ exploitable to identify LN recurrence site. A large retrospective multi-institutional study comparing stereotactic body radiotherapy (SBRT) to extended nodal radiotherapy (ENRT), for prostate cancer LN relapse observed better metastasis-free survival (MFS) in patients with one LN at recurrence after ENRT.^16^ No similar studies exist, and the question is also of interest for gynecological tumors, with a different, more aggressive biology. We compared outcomes and toxicity in two groups of patients affected by primary gynecological LN recurrence, treated with SBRT on the FDG PET/CT positive LNs versus ENRT on the interested chain with simultaneous integrated boost (SIB) to the FDG PET/CT positive LN.

## Material and methods

This retrospective study was performed according to the Helsinki Declaration. Informed consent for treatment and disease-related information publication was obtained from all participants. The Ethics Committee 1 of the Lombardy region approved the study (register number 43-2024, ClinicalTrials.gov Identifier NCT06306170).

### Endpoints of the study

The main objective of our study was to analyze the disease control in terms of local relapse-free survival (LRFS), regional relapse-free survival (RRFS), distant metastases-free survival (DMFS), disease-free survival (DFS), and overall survival (OS) in gynecological cancer patients presenting disease recurrence only at the lymph node level treated with two possible radiation therapies. Therapeutic strategies included extended nodal radiotherapy (ENRT), which delivers a prophylactic dose to the affected lymph node areas (pelvic, para-aortic, mediastinal) thus also considering the circulating neoplastic cells (not observable by PET), and stereotactic irradiation (SBRT) of the PET-positive lymph node(s) only, to treat exclusively the significant disease burden. Secondary endpoints were acute and late toxicity of the two therapeutic strategies, and the identification of factors influencing outcomes in our cohort of patients.

### Inclusion and exclusion criteria

Records of gynecologic patients treated in our institution since 2005 with intensity-modulated radiotherapy (IMRT), image-guided radiotherapy (IGRT), or SBRT, and with FDG PET/CT guided treatment planning were reviewed; only patients at first salvage radiotherapy for LN relapses were considered. Patients with a second relapse at the LN level, as well as those with bone or visceral metastases, were excluded.

The choice of radiation treatment type was made according to the judgment of the treating physician, based on the knowledge at the time of prescription and taking disease burden and location into account.

### Treatment, simulation, dose prescription, planning, follow-up

Patients with no other reasonable therapeutic alternatives (surgery or systemic therapy), whose cases were discussed in the multidisciplinary meeting, received the indication of salvage radiotherapy. Median interval between the last oncological treatment and salvage radiotherapy was 8.5 (1.6-55.0) months. No concomitant or adjuvant systemic treatment was planned. Further oncological treatment was prescribed only in case of relapse after radiotherapy.

**Figure 1.**
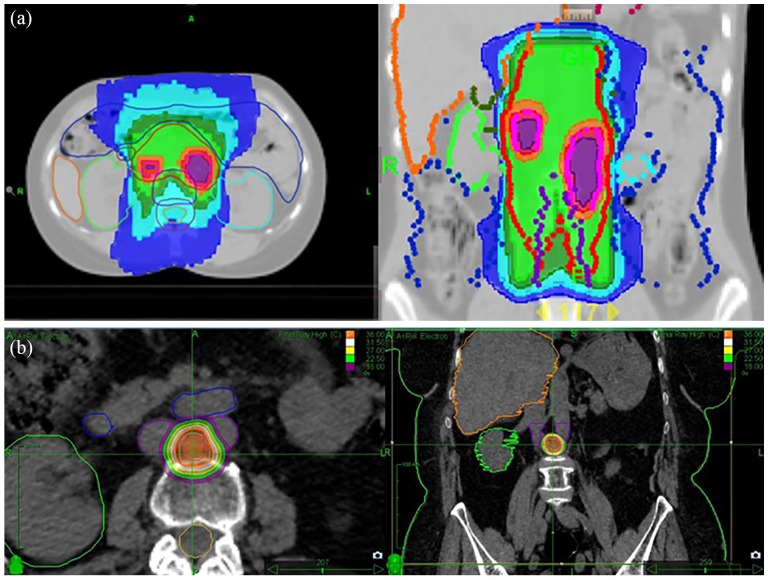
a. 21-year-old patient with bilateral ovarian endometrioid adenocarcinoma, operated on and treated with chemotherapy in 2008, relapsed in 2010, treated with two lines of chemotherapy with stable disease, irradiated in 2011 to the para-aortic lymph node areas (ENRT) at TD of 50.4 Gy/ 28 fr with SIB to PET positive LNs at TD of 62.5 Gy (BED = 76.44 Gy) with controlled disease, and with no further relapse. During treatment she presented G2 nausea, vomiting, fatigue, and later occasional G1 anemia for several years. b. 75-year-old patient with transitional cell ovarian adenocarcinoma operated on and treated with chemotherapy in 2006, relapsed, treated with multiple lines of chemotherapy, then Olaparib from 11.2016 to 01.2019, with progressive disease. Irradiated on the inter-aorto-caval lymph node at TD of 36 Gy / 3 fr (BED 79.2 Gy), she presented excellent local control (without relapse at almost five years), but progression both loco-regional para-aortic, and peritoneal at three months after SBRT. She had no acute or late toxicity.

Simulation CT was performed in supine position, using immobilization systems specifically adapted for the site to be treated, and treatment machine. CT section thickness was 3 mm for helical TomoTherapy (TomoTherapy, Accuray, Maddison, WI, USA) or RapidArc (Varian, Palo Alto, CA, USA) treatments and 1.25 mm for CyberKnife M6 (Accuray, Sunnyvale, CA, USA) treatments. Nodal gross tumor volume (GTVN), of the PET/CT positive LN was defined based on FDG PET/CT for both groups. Nodal planning target volume (PTVN) was defined by isometrically expanding GTVN by 5 mm for ENRT treatments, and 3 mm for SBRT. Furthermore, for ENRT treatments, the involved LN chain was defined as CTV (Clinical Target Volume) and expanded by 7 mm, to generate the chain PTV (LNPTV). Median dose delivered to LNPTV in the ENRT group was 50.4 (40.05-50.4) Gy in a median of 28 (15-28) fractions, with SIB up to a median of 61.6 (44.4-65.5) Gy, corresponding to a BED (Biological Effective Dose) of 76.5 (IQR: 74.4-78.7) Gy. In the SBRT group, the median dose delivered was 40 (30-63.2) Gy with a median of 5 (3-10) fractions and a median BED of 72 (IQR: 59.5-75.6) Gy. The differences in volume and prescribed dose between ENRT and SBRT are evident in Figure 1a and 1b.

Patients were evaluated every three to four months in the first year, then every six months through the fifth year, with blood chemistry tests, CT/magnetic resonance (MRI)/PET and gynecological examination. Maximum values of acute and late toxicity were classified according to Common Terminology Criteria of Adverse Events (CTCAE), v5.0.

### Statistical analysis

To compare treatment groups with respect to demographic and clinical variables Mann-Whitney and Fisher's exact tests were applied for quantitative and qualitative variables, respectively. Local relapse-free survival (LRFS) was defined as period from radiotherapy end to local relapse or death; regional relapse-free survival (RRFS) as the period from the treatment end to regional relapse, in the nodal chain treated with ENRT, or the chain of which the SBRT treated node was part, or patient's death; distant metastasis-free survival (DMFS) as the time from treatment end to distant recurrence, or death; disease-free survival (DFS) as time from treatment end to any event (local, regional, distant relapse, death, whichever occurred first); overall survival (OS) as time from radiation end to patient death.

The reverse Kaplan-Meier method was used to compute median follow-up and interquartile range. The Kaplan-Meier method was used to estimate survival curves, and the log-rank test to compare survival curves between groups.

Survival Trees were implemented to identify new cut-offs for treatment volumes and doses distinguishing different prognostic groups.^17^ To avoid overfitting, the minimum number of observations in any terminal node was limited to 10. Cox regression models were estimated to identify factors associated with survival outcomes. Analyses were performed with R statistical software (R Core Team 2023, version 4.3.1). In all analyses, the significance level was set at 5%.

## Results

### Demographic and treatment data

Fifty-eight patients treated from February 2007 to April 2023 in our institution with intensity-modulated radiotherapy (IMRT), image-guided radiotherapy (IGRT), or SBRT for pelvic or extra-pelvic first LN recurrence from gynecologic tumors were identified. The patients were divided into two groups: 23 (ENRT group) underwent ENRT to the interested LN chain, and SIB to the FDG PET/CT positive LNs; 35 (SBRT group) underwent SBRT to LN recurrence. Table 1 summarizes patient characteristics.

**Table 1.** The characteristics of the patients treated with radiotherapy to the entire involved lymph node area and simultaneous integrated boost to PET positive lymph-nodes (ENRT+SIB) vs. only positive lymph nodes (SBRT).

Variables		ENRT (n=23)	SBRT (n=35)	*p*-value
Age at diagnosis (median [IQR])		60.44 [47.94, 67.02]	55.23 [51.79, 71.70]	0.489
Age at salvage RT (median [IQR])		65.93 [52.55, 71.10]	67.25 [56.61, 74.60]	0.404
Histology (%)	Endometrium	9 (39.1)	9 (25.7)	0.168
	Cervix	2 (8.7)	10 (28.6)	
	Ovary	12 (52.2)	16 (45.7)	
FIGO stage (%)	I	6 (26.1)	9 (25.7)	0.590
	II	2 (8.7)	8 (22.9)	
	III	11 (47.8)	13 (37.1)	
	IV	4 (17.4)	5 (14.3)	
Surgery for primary tumor (%)	no	1 (4.3)	5 (14.3)	0.386
	yes	22 (95.7)	30 (85.7)	
RT for primary tumor (%)	no	18 (78.3)	22 (62.9)	0.257
	yes	5 (21.7)	13 (37.1)	
Chemotherapy for primary (%)	no	3 (13.0)	8 (22.9)	0.499
	yes	20 (87.0)	27 (77.1)	
PFS (median [IQR])		19.01 [10.91, 30.36]	18.58 [9.95, 33.67]	0.855
Surgery at first relapse (%)	no	21 (91.3)	25 (71.4)	0.099
	yes	2 (8.7)	10 (28.6)	
RT at first relapse (%)	no	16 (69.6)	17 (48.6)	0.175
	yes	7 (30.4)	18 (51.4)	
CT at first relapse (%)	no	6 (26.1)	13 (37.1)	0.410
	yes	17 (73.9)	22 (62.9)	
Previous RT to same volume (%)	no	20 (87.0)	29 (82.9)	1.000
	yes	3 (13.0)	6 (17.1)	
Nr of metastases treated (%)	1	8 (34.8)	**29 (82.9)**	**<0.001**
	2	1 (4.3)	4 (11.4)	
	3	3 (13.0)	2 (5.7)	
	4	3 (13.0)	0 (0.0)	
	⩾6	**8 (34.8)**	0 (0.0)	
Localization of LN metastases	Pelvic	3 (13%)	11 (31.4%)	0.185
	Para-Aortic	15 (65.2%)	15 (42.9%)	
	Extra-Abdominal	5 (21.8%)	9 (25.7%)	
GTVN (median [IQR])		24.40 [9.35, 42.25]	4.30 [2.18, 10.42]	**<0.001**
PTVN (median [IQR])		89.40 [36.05, 124.45]	10.20 [6.10, 25.70]	**<0.001**
BED (median [IQR])		76.45 [74.40, 78.67]	72.00 [59.50, 75.60]	**0.006**

IQR, interquartile range; RT, Radiotherapy; FIGO, International Federation of Gynecology and Obstetrics; PFS, progression-free survival; LN, lymph node; CT, chemotherapy; GTVN, Nodal Gross Tumor Volume; PTVN, Nodal Planning Target Volume; BED, biologically effective dose.

Values in bold indicate statistically significant differences.

At the first LN recurrence, for which the patients underwent the salvage treatment, the largest group (47.8%) of the 23 patients with 131 LNs treated with ENRT presented >3 positive LNs. In the SBRT group (35 patients with 47 LNs), most cases (82.9%) presented recurrence in a single LN. In the ENRT group, 13.0% of cases were previously irradiated on the same site, while in the SBRT group, 17.1%.

In the comparison of treatment volumes, the total volume of GTVN and PTVN of individual LNs receiving SIB in the ENRT group, and SBRT in the other, were used. Median GTVN for SIB was 24.4 cc and median PTVN 89.4 cc, while for SBRT, median GTVN and PTVN were 4.3 cc and 10.2 cc, respectively (p<0.05). In the ENRT group, SIB was not prescribed for four patients, who received a uniform dose over the entire disease volume because, in two cases, given the significant disease extension (precluding acceptable dose distribution and target coverage) SIB would have exceeded dose limits for neighboring organs at risk (OAR). In two other cases, SIB was not prescribed as the patients had received chemotherapy prior to radiotherapy (leading to a reduction in metabolic activity and size of PET-positive LNs).

### Endpoint analysis results

Median follow up for the ENRT group was 81.1 months (IQR: 48.5;117.2), for SBRT 37.0 months (IQR: 21.3;58.4).

Given the different follow-ups in the two groups, the estimate was made at 36 months, considered sufficient to express the outcomes for OMD, and higher than most median follow-ups published to date. Estimated 36-month LRFS was 90.2% in ENRT group and 82.6% in SBRT group (p=ns); RRFS was 69% vs 63.4%, respectively (p=ns). DMFS was 26.1% vs 44.3% (p=ns); and DFS was 26.1% vs 32.2%, respectively (p=ns). Thirty-six-month OS was 73.7% in the ENRT group and 60.4% in the SBRT group, and globally not statistically different in the two treatment groups (logrank test, p= 0.29).

Differences in LRFS, RRFS, DMFS, DFS and OS were also evaluated for patient groups classified by primary tumor (endometrial, cervical, ovarian) generating the metastases. No statistically significant difference was observed between the groups for any survival outcome.

**Figure 2.**
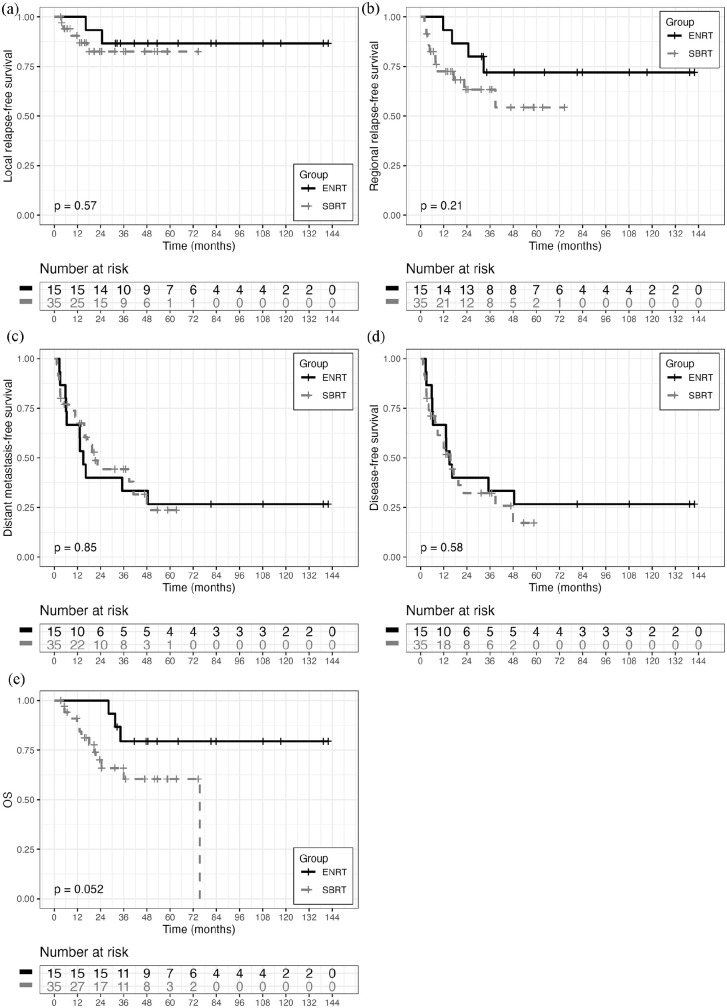
a. Overall survival, b. local relapse-free survival, c. regional relapse-free survival, d. distant relapse-free survival, e. disease-free survival, in patients with up to 5 LN metastases in ENRT+SIB vs SBRT group.

The analysis was repeated eliminating patients with >5 metastases (not oligometastatic) from the ENRT group, to better homogenize the two groups: no statistically significant differences were observed (See Figure 2 a-e).

Acute toxicity depended on treatment site and included: diarrhea, nausea and vomiting, dysphagia, esophagitis and gastralgia, cough, asthenia, with CTCAE v5.0 maximum grade 2 (G2). Two patients presented hematological toxicity (anemia and leukopenia). Only one presented G3 skin erythema. For late toxicity, only one patient presented G3 toxicity (pelvic bone fracture) after pelvic radiotherapy. A significant association emerged between treatment group and genitourinary (GU), gastro-intestinal (GI, including esophageal for mediastinal treatments), and other acute toxicities (cutaneous, hematological, asthenia), with higher toxicity grade in the ENRT group, as seen in Table 2, while no significant association between treatment group and late toxicity was observed.

**Table 2.** Toxicities in ENRT+SIB and SBRT groups.

Toxicity	Grade	ENRT (n=23)	SBRT (n=35)	p-value
Acute GU (%)	0	18 (78.3)	35 (100.0)	**0.007**
	1	3 (13.0)	0 (0.0)	
	2	2 (8.7)	0 (0.0)	
Acute GI (%)	0	11 (47.8)	29 (82.9)	**0.021**
	1	5 (21.7)	3 (8.6)	
	2	7 (30.4)	3 (8.6)	
Acute other (%)	0	16 (69.6)	32 (91.4)	**0.033**
	1	5 (21.7)	1 (2.9)	
	2	1 (4.3)	2 (5.7)	
	3	1 (4.3)	0 (0.0)	
Late, all toxicities (%)	0	19 (82.6)	33 (94.3)	0.193
	1	3 (13.0)	1 (2.9)	
	2	0 (0.0)	1 (2.9)	
	3	1 (4.3)	0 (0.0)	

Values in bold indicate statistically significant differences.

There was no significant difference between groups with G0+G1 (44 patients) vs G2+G3 (14 patients) toxicity, considering maximum acute toxicity experienced, with respect to target volume (p-value= 0.07).

**Figure 3.**
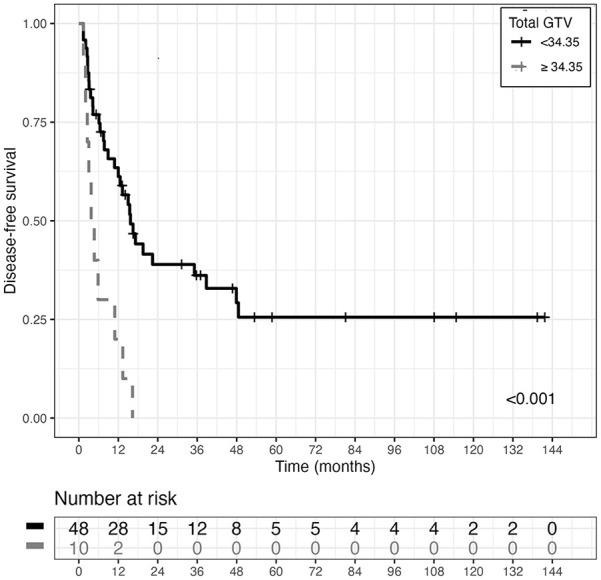
Disease-free survival curves (DFS) estimated with the Kaplan-Meier (KM) method, in the two GTV (Gross Tumor Volume, nodal) groups of patients derived using ST analysis cut-off value (p-value associated with logrank test is also reported).

The best GTV cut-off value for disease control, identified on the basis of the Survival Tree analysis (ST), was 34.35 cc (see Figure 3). A total GTV<34.35 cc was present in 65.2% of ENRT patients and 94.3% of SBRT patients.

Expansion from GTVN to the PTVN was larger for IGRT-IMRT treatments with ENRT and SIB, compared to SBRT treatments. Therefore, predictive PTVN for treatment response was also identified, and was 81.3 cc(p=0.011). Only 47.8% of ENRT patients presented a PTV<81.3 cc vs 100% of SBRT patients.

**Figure 4.**
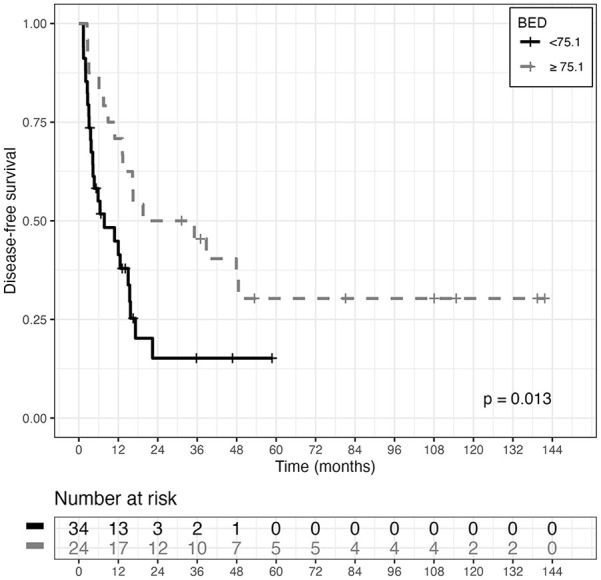
Disease-free survival curves (DFS) estimated with the Kaplan-Meier (KM) method, in the two BED (Biological Effective Dose) groups of patients, derived using ST analysis cut-off value (p-value associated with logrank test, is also reported).

The BED cut-off identified through the survival tree is 75.1 Gy (p=0.013), and 34.8% of ENRT-SIB patients were treated with a BED <75.1 Gy, vs 74.3% of SBRT patients (See Figure 4).

Age at diagnosis, histology (ovarian, endometrial, cervical), FIGO stage, PFS (interval between primary tumor diagnosis and metastasis), GTV (⩾34.35 vs <34.35), PTV (⩾81.3 vs <81.3) and BED (<75.1 vs ⩾75.1) were examined as potential risk factors for DFS in a univariate Cox regression analysis. Larger tumor volume: GTV (HR=3.6333; 95%CI: [1.7046,7.7441]; p=0.0008); and PTV (HR=2.4242: 95%CI: [1.2015,4.8914]; p=0.0134), and lower BED (HR=2.255; 95%CI: [1.1707, 4.3439]; p=0.0151), emerged as risk factors significantly associated with DFS.

When entering all the abovementioned covariates into a Cox multivariate model for DFS, but not PTV (associated with GTV), in the model selected, using a backward approach, only GTV (HR=4.6192; 95%CI: [2.088-10.2188]; p=0.0002) and BED (HR=2.7002; 95%CI: [1.3797-5.2847]; p=0.0037) were retained as significant variables.

## Discussion

Two treatment modalities for LN recurrence are available: ENRT and SBRT. ENRT studies are older and small (12-50 patients). Grigsby et al.^1^ retrospectively analyzed 20 para-aortic LN relapsed cervical cancer patients reporting hydronephrosis, lower extremity edema and neuropathic sciatic pain, treated with conventional radiotherapy (RT); all died within two years from disease progression. Longer interval to relapse and a total dose >45 Gy improved median survival. In 14 patients with para-aortic LN relapse, all seven with Grisgby’s symptoms treated with <45 Gy concurrent radio-chemotherapy (CCRT), were dead within 1.5 years, while the seven patients without symptoms, treated with full-dose, had a five-year OS of 100%.^2^ Predictive factors for local failure identified in 38 patients with endometrial adenocarcinoma treated with ENRT to pelvic or para-aortic LN were: previous chemotherapy, <64.7 Gy SIB, grade 3 tumor, and <1 year to recurrence.^3^ In ovarian cancer patients with LN or isolated extra-LN recurrence, better response to radiotherapy vs chemotherapy (64% vs 16.7%) was observed, with lower toxicity and no significant difference in OS and DFS.^18^ Of the 26 cervical cancer patients with para-aortic relapse, only those treated with CCRT to para-aortic LNs presented long-term DFS; patients treated with chemotherapy or radiotherapy alone, or untreated, died.^19^ In 22 LN relapses (including 10 supraclavicular LNs), with median 31.2 month follow-up, five-year DFS and OS was 32.7% and 30.7%, respectively.^20^ Patients with ⩽8 ng/dl squamous cell carcinoma antigen and >18 months to LN recurrence, had better OS. Treatment failure occurred in 63.6% of patients, and three patients developed G3 toxicity.^20^ With 2D hyperfractionated radiotherapy (two daily 1.2 Gy fractions to total dose of 60 Gy) and concurrent chemotherapy for para-aortic LN recurrence in 12 cervical carcinoma patients, three-year OS was 19% and relapses >24 months from primary treatment had better median survival.^21^ Two patients had G3-4 hematologic toxicity, 50% G2 nausea.^21^ The largest published study analyzed 50 patients with para-aortic LN recurrence: 24 had 1-2 metastases, and 26 had ⩾3. Three-year OS was 85.7% for CCRT, 66.7% for surgery, 48.8% for chemotherapy, 41.3% for radiotherapy, 0 for best supportive care (p=0.014).^22^ Five-year OS was 37.5% in a recent study of 20 cervical cancer patients with LN recurrence treated with CCRT or radiotherapy alone to para-aortic or pelvic chain. Three-year OS for patients with recurrence ⩽9 months after primary treatment was 20%, and 70% for later recurrences.^23^

SBRT studies are more recent. MITO RT1, analyzing results based on treated lesions rather than patients (as in our analysis), observed a 24-month LRFS of 81.9% for patients with oligometastatic/progressive disease from ovarian tumors.^4^ In the MITO-RT2/RAD study on oligometastases from cervical cancer,^5^ local control was 94.4% with median follow-up of 14.5 months, and 94.5%, with the same median follow-up for oligometastases from endometrial carcinoma.^6^ Similar evidence emerged from previous smaller studies.^24,25^ These results obtained recently with modern techniques are superior to those obtained with ENRT in the 2000s. In fact, Grigsby et al,^1^ in patients with para-aortic LN recurrence from cervical cancer, observed median survival of 8.7 months in the entire group.

In our patient cohort estimated 36-month LRFS was 90.2% for ENRT and 82.6% for SBRT in line with modern series.

MITO RT1 study identified a group of disease and therapy characteristics predictive of better response to SBRT for ovarian cancer: age <70 years, LN metastases, disease volume (PTV < 18cc) and BED delivered (cut-off: 70 Gy).^4^ Patients in our study had greater median GTV, and median BED delivered was slightly higher in both groups. The Cooperative Study of the Korean Radiation Oncology Group (KROG 14-11) of SBRT for recurrent oligometastatic uterine cervix cancer, in a patient group with numerous LN metastases, observed a 32.9% five-year OS.^26^ Two and five-year LRFS were 82.5 and 78.8%, respectively, and BED>90 Gy(p=0.072), BED>69 Gy(p=0.059), and longer DFS predicted marginally superior LC.^26^

The toxicity found for our stereotactic treatments, as in the multi-institutional MITO studies,^4-6^ was low. ENRT toxicity was as high as G2 in 30% of patients in the Lee et al. study^18^ - still significantly lower than the toxicity with chemotherapy. In another study toxicity was higher, reporting G⩾3 in 21% of patients, due to large treatment volumes and associated chemotherapy in 55% of patients.^3^ In our study only one acute G3 toxicity (skin erythema), and one late G3 toxicity (bone fracture) were detected.

An important limitation of our study, as of all published ENRT studies for gynecological cancer LN relapses, is its retrospective nature in a limited, single-institutional sample. Compared to recent SBRT studies, however, our analysis has the advantage of including only LN recurrences, where an extensive treatment including the entire LN chain could offer a benefit, given the extension by circulating tumors cells between LN.^16,27^ To overcome the main limitations (statistically different follow-ups and number of lymph-nodes) the analysis was performed limiting the follow-up to 36 months, and repeated, limiting the total number of lymph-nodes to four.

To the best of our knowledge, this is the first study reporting results in a cohort treated with both ENRT and SBRT for LN relapses, in gynecological tumors. This allows us to make an initial contribution in evaluating the benefits in LC, OS and DFS for ENRT vs SBRT in LN recurrences. As in the MITO RT studies, failure was mainly distant, suggesting that hematogenous diffusion predominates over lymphatic. In prostate cancer, however, extensive treatments seem to lead to better disease control.^16,28,29^ Given the results achieved with similar samples of prostate cancer patients^28,29^ we are led to hypothesize that the impact of the extensive treatment is lower in gynecological patients. The results may be partially due to the impact of 18F-FDG PET/CT, which correctly selected the significant burden of disease.^12-15,30,31^

The choice of the type of treatment was made according to the judgment of the treating physician, taking disease burden into account. Patients treated with SBRT had a lower number of LN involved: only one LN for 82.9% of patients treated with SBRT vs 34.8% for ENRT patients. The patients with more than six positive LN represent one third of patients treated with ENRT, while no patient treated with SBRT had more than three synchronous lesions. In consideration of the similar DFS achieved, it could be hypothesized that ENRT compensated for the greater disease burden, allowing good local and regional control. This observation represents further data in support of the previous hypothesis: good local and regional control had no significant impact on distant disease control or OS, a finding confirmed by multicenter MITO-AIRO Gyn studies,^4-6^ and early ENRT studies.^1-3,18-23^ No statistically significant differences were observed when the analysis was limited to OMD (⩽5 LNs).

Another necessary observation concerns the prescribed doses. Although a 70 Gy BED is considered sufficient^4^; SABR COMET^8^ demonstrated an increased survival with ablative doses, in patients with different primary tumors (prostate, breast, lung). This BED was not reached even in the KROG 14-11 trial.^26^ Evidence of a dose effect also in our cohort indicates the need to explore, when safely deliverable, more aggressive treatments on positive LNs.

Radiotherapy has enabled treated patients to achieve a systemic therapy-free interval, allowing, in some cases, the subsequent reintroduction of systemic therapy upon relapse.^4-6^ Alternatives to radiotherapy are: the most frequent second look surgery, hadron therapy or the least used radiofrequency ablation.^32-35^ Quality of life plays an increasingly important role in the selection of oncological treatments and, although not investigated in this particular situation of LN recurrence, surgery can have a significant impact in gynecological as well as other tumor locations.^36-38^ This has paved the way for the use of radiotherapy in LN recurrence, but the extension of the radiotherapy fields remains to be investigated.

## Conclusions

We can reasonably consider these two treatment modalities as valid therapeutic options in LN oligometastatic patients, obtaining good disease control with acceptable toxicity. Stereotactic treatments would be preferable for small volumes. Larger volumes will probably require ENRT. Prospective studies with adequate samples are necessary to identify the treatment field extension and BED values necessary for positive results in gynecological tumor LN recurrences, for which (due to their specific biology), it is impossible to automatically apply results deriving from other tumor types.
